# Safety and Efficacy of Anti-PD-1/PD-L1 Inhibitors Compared With Docetaxel for NSCLC: A Systematic Review and Meta-Analysis

**DOI:** 10.3389/fphar.2021.699892

**Published:** 2021-08-12

**Authors:** Long Ma, Gang Jin, Keying Yao, Yi Yang, Ruitong Chang, Wenhao Wang, Jiawei Liu, Zijiang Zhu

**Affiliations:** ^1^ The First Clinical Medical College of Gansu University of Chinese Medicine (Gansu Provincial Hospital), Lanzhou, China; ^2^ Department of Thoracic Surgery, Gansu Provincial Hospital, Lanzhou, China; ^3^ The Second Clinical Medical College of Lanzhou University, Lanzhou, China

**Keywords:** anti-PD-1/PD-L1 inhibitors, DOCETAXEL, systematic review, meta-analysis, NSCLC

## Abstract

**Objective:** To evaluate the efficacy and safety of anti-PD-1/PD-L1 Inhibitors versus docetaxel for non-small cell lung cancer by meta-analysis.

**Methods:** Randomized controlled trials (RCTs) about anti-PD-1/PD-L1 Inhibitors versus docetaxel on the treatment of NSCLC were searched in CNKI, WF, VIP, PubMed, EMBASE, Cochrane Library, and Web of Science databases. Two reviewers independently screened literature, extracted data and evaluated the risk of bias of eligible studies. Meta-analysis was performed by RevMan5.3 software.

**Results:** Compared with the use of docetaxel chemotherapy for NSCLC, the overall survival and progression-free survival of the anti-PD-1/PD-L1 Inhibitors regimen are better [overall survival: (HR= 0.73, 95%CI:0.69∼0.77, P<0.00001], progression-free survival: (HR= 0.89, 95%CI:0.83∼0.94, P<0.00001]), and lower incidence of treatment-related grade 3 or higher adverse events ([OR=0.20, 95% CI: 0.13∼0.31, P<0.00001]).

**Conclusion:** Compared with the docetaxel chemotherapy regimen, the anti-PD-1/PD-L1 Inhibitors has certain advantages in terms of efficacy and safety. The results still need to be confirmed by a multi-center, large sample, and high-quality research.

## Introduction

Lung cancer is one of the most common malignant tumors in the world, originating in the trachea, bronchus and lung (Bade and Dela Cruz, 2020). Its morbidity and mortality rank first among all tumors, which seriously threaten the health and life quality of patients (Romaszko and Doboszyńska, 2018). Lung cancer is mainly divided into two types: small cell lung cancer (SCLC) and non-small cell lung cancer (NSCLC), of which NSCLC accounts for 85% (Nasim et al., 2019). Due to the low early diagnosis rate, most patients are already in the late stage at the time of diagnosis, and the 5-years survival rates of patients with locally late stage or distant metastasis are only 26 and 4%, respectively (The Lancet, 2019). Patients usually have common symptoms such as cough, chest tightness, chest pain, and difficulty breathing (Hoy et al., 2019). Currently, chemotherapy drugs containing platinum and docetaxel are the standard chemotherapy regimens for NSCLC (Sakaguchi et al., 2020). The main targe of docetaxel is microtubules, its pharmacological mechanism is to promote tubulin polymerization and inhibit microtubule depolymerization, thus inhibit tumor cell division and growth. In addition, docetaxel can also promote the apoptosis of tumor cells by up-regulating BIM and enhance the expression of TRAIL in clinical treatments. However, there have low effective rate, short survival period, and more serious adverse reactions (An et al., 2019). For NSCLC, there is still need to develop its therapeutic drugs.

Despite the application of molecular targeted therapies, anti-angiogenesis and new chemotherapeutic drugs, and significant improvements in therapeutic effects, the prognosis of most patients is still poor (Jonna and Subramaniam, 2019). Research in recent years has recognized that tumor cells can induce immune tolerance in the tumor microenvironment by blocking the immune checkpoint of cytotoxic T cells (Suresh et al., 2018). For the understanding of tumor immune evasion mechanism, immunotherapy has become an important part of tumor treatment, and immune checkpoint inhibitor therapy based on PD-1/PD-L1 antibody has become one of the standard treatment methods (Sui et al., 2018). PD-1/PD-L1 immune checkpoint inhibitor uses the body’s own immune system to kill tumor cells (Li et al., 2019). The interaction of PD-1/PD-L1 can inhibit T cell responses, promote the differentiation of CD4^+^ T cells into T regulatory cells, induce tumor-specific T cell apoptosis (Liu et al., 2020). PD-1/PD-L1 can combine to activate the PD-1/PD-L1 signaling pathway, thereby inhibiting the immune activity of T cells, causing tumor immune escape, leading to tumor occurrence and development (Chae et al., 2018). Blocking the PD-1/PD-L1 signaling pathway can reverse the tumor immune microenvironment and enhance the endogenous anti-tumor immune effect (Hersom and Jørgensen, 2018). In this study, the method of meta-analysis was used to systematically evaluate the efficacy and safety of PD-1/PD-L1 and docetaxel-containing chemotherapy regimens, in order to provide a basis for clinical work to a certain extent.

## Methods

This study was performed in accordance with Preferred Reporting Items for Systematic Reviews and Meta-Analyses (PRISMA).

### Data Sources and Searches

CNKI, VIP, WF, PUBMED, embase, Web of Science, and Cochrane Library database were systematically searched to identify randomized controlled trials (RCTs) from inception to February, 2021. The search strings consisted in a combination of the following keywords: “anti-programmed cell death 1”, “anti-PD-1”, “anti-programmed cell death ligand 1”, “anti-PD-L1”, “docetaxel”, “non-small cell lung cancer”, “NSCLC”, “Randomized controlled trials”, “RCTs”.

### Inclusion Criteria

Studies eligible to be included in this study were required to meet the following criteria: 1) Population: patients with NSCLC; 2) Intervention and comparison: anti-PD-1/PD-L1 was compared with docetaxel; 3) Outcomes: overall survival (OS), progression-free survival (PFS), treatment-related grade 3 or higher adverse events.

### Exclusion Criteria

We excluded studies without original data, meta‐analyses, animal‐based studies, abstracts only, studies with duplicated data, case reports, case series, and observational studies.

### Study Selection

After removal of duplicates, two independent researchers screened all titles and abstracts. They obtained full texts and performed further screening when studies were identified as potentially eligible. Any disagreement was settled by consensus among all authors.

### Data Extraction

For each eligible study, pairs of reviewers, following training exercises, extracted data independently using a standardized data extraction form. Reviewers collected information on author, publication date, country, study design, overall survival (OS), progression-free survival (PFS), treatment-related grade 3 or higher adverse events. Any disagreement was settled by consensus among all authors.

### ROB Assessment

ROB was assessed by two independent reviewers using the Cochrane Collaboration’s tool for ROB assessment.

### Statistical Analysis

We performed statistical analyses using RevMan5.3. We used risk ratios and 95% confidence intervals to assess outcomes, and considered a *p* value less than 0.05 to be statistically significant. We assessed heterogeneity using the *I* ^2^ test. If significant heterogeneity was not present (*I* ^2^ < 50%), we used fixed effects models to pool outcomes; we used random effects models when significant heterogeneity was present (*I* ^2^ ≥ 50%).

## Results

### Eligible Study Characteristics

**FIGURE 1:**
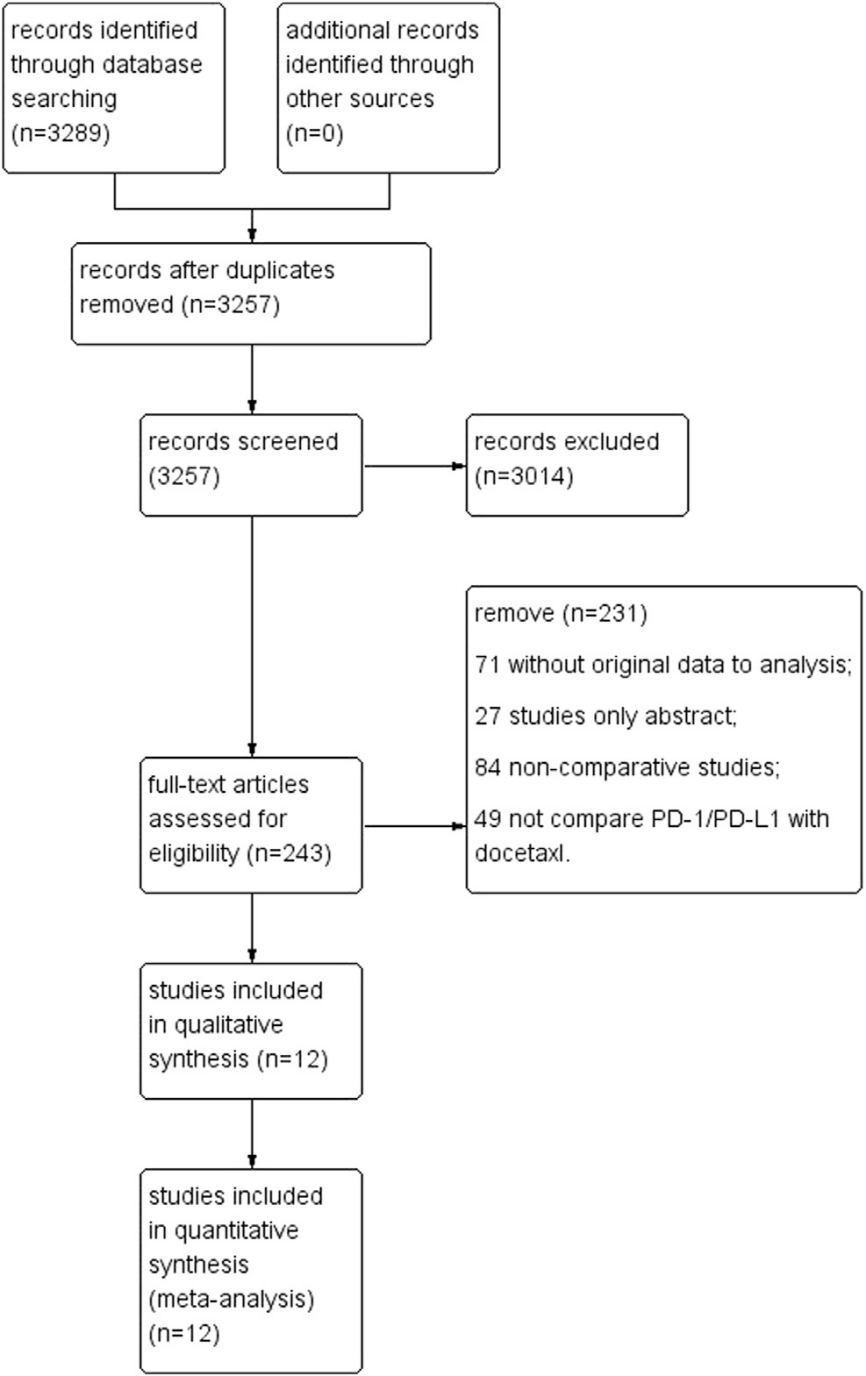
Flow chart of study selection.

After screening, we finally included 12 RCTs for Systematic review and Meta-analysis. Among them, 4,684 patients were treated with PD-1/PD-L1, and 4,610 patients were treated with docetaxel. The literature screening process is shown in Figure 1 (Flow chart of study selection), the basic characteristics are shown in Table1 (Basic characteristics of the study).

**TABLE 1:** Basic characteristics of the study.

Author	Year	Patients	Treatment arms	Overall survival [HR(95%CI)]	Progression-free survival [HR(95%CI)]	Treatment-related grade 3 or 4 adverse events	Study design
Borghaei et al. (2015)	2015	292	Pembrolizumab 200 mg q^3^w	0.73(0.59–0.89)	0.92(0.77–1.10)	30	RCT
		290	Platinum-based chemotherapy			144	
Brahmer et al. (2015)	2015	135	Nivolumab 3 mg/kg, q^2^w	0.59(0.44–0.79)	0.62(0.47–0.81)	9	RCT
		137	Docetaxel 75 mg/m^2^, q^3^w			71	
Fehrenbacher et al. (2016)	2016	144	Atezolizumab 1200 mg, q^3^w	0.73(0.53–0.99)	0.94(0.72–1.23)	57	RCT
		143	Docetaxel 75 mg/m^2^, q^3^w			71	
Herbst et al. (2016) 10 mg	2016	346	Pembrolizumab 10 mg/kg q^2^w	0.61(0.49–0.76)	0.79(0.66–0.95)	55	RCT
		343	Docetaxel 75 mg/m^2^ q^3^w			109	
Rittmeyer et al. (2017)	2017	425	Atezolizumab 1200 mg q^3^w	0.73(0.62–0.87)	0.95(0.82–1.10)	90	RCT
		425	Docetaxel 75 mg/m^2^ q^3^w			247	
Barlesi et al. (2018)	2018	396	Avelumab 10 mg/kg q^2^w	0.90(0.72–1.12)	1.01(0.80–1.28)	39	RCT
		396	Docetaxel 75 mg/m² q^3^w			180	
Fehrenbacher et al. (2018)	2018	613	Atezolizumab 1200 mg q^3^w	0.80(0.70–0.92)	0.96(0.85–1.08)	91	RCT
		612	Docetaxel 75 mg/m^2^ q^3^w			245	
Vokes et al. 2018	2018	427	Nivolumab, 3 mg/kg, q^2^w	0.70(0.61–0.81)	0.80(0.69–0.92)	44	RCT
		427	Docetaxel, 75 mg/m^2^, q^3^w			/	
Von Pawel et. al (2019)	2019	398	Atezolizumab 1200 mg q^3^w	0.75(0.64–0.89)	/	64	RCT
		376	Docetaxel 75 mg/m^2^ q^3^w			/	
Horn et al. 2017	2017	427	Nivolumab, 3 mg/kg, q^2^w	0.72(0.62–0.84)	/	19	RCT
		427	Docetaxel, 75 mg/m^2^, q^3^w			/	
Barlesi et al (2019)	2019	312	Pembrolizumab 2 mg/kg q^3^w	/	/	/	RCT
		266	Docetaxel 75 mg/m^2^ q^3^w			/	
Bordoni et al. (2018)	2018	425	Atezolizumab 1200 mg q^3^w	0.73(0.62–0.87)	/	/	RCT
		425	Docetaxel 75 mg/m^2^ q^3^w			/	

Progression-Free Survival: the time between the onset of treatment for a cancer disease and the observation of disease progression or death from any cause.treatment-related grade 3 or 4 adverse events: severe Granulocytopenia, CIA, Fatigue, etc.q^2^w:2 weeks using a time, q^3^w:3 weeks using a time: RCT: randomized controlled trial.

### Quality Assessment

**FIGURE 2:**
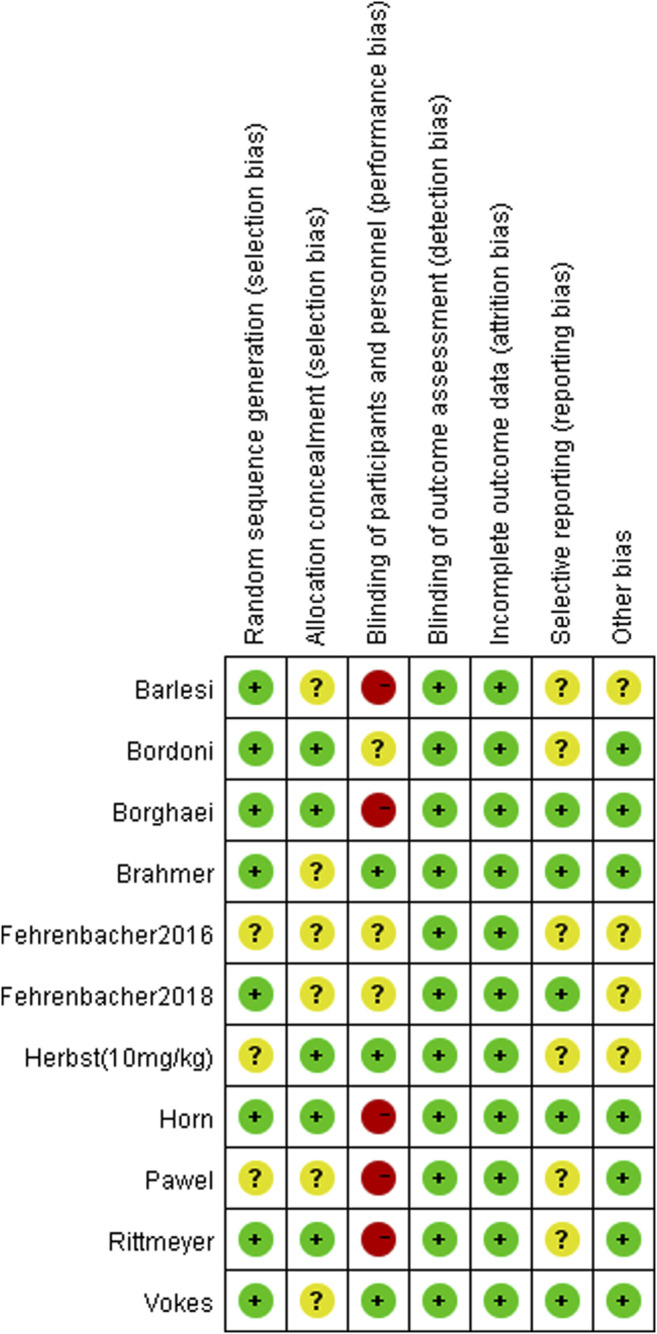
Risk of bias summary.

The results of the quality assessment are shown in Figure 2 (Risk of bias summary). Most studies had a low risk of bias. Random sequence generation was not found in three studies (Fehrenbacher et al., 2016; Herbst et al., 2016; von Pawel et al., 2019), and some studies did not clearly report concealment (Brahmer et al., 2015; Fehrenbacher et al., 2016; Fehrenbacher et al., 2018; Vokes et al., 2018; von Pawel et al., 2019; Barlesi et al., 2019). The blinding of participants was explicitly reported in three studies (Brahmer et al., 2015; Herbst et al., 2016; Vokes et al., 2018). Furthermore, some studies did not clearly report selective reporting (Fehrenbacher et al., 2016; Herbst et al., 2016; Rittmeyer et al., 2017; von Pawel et al., 2019; Barlesi et al., 2019; Bordoni et al., 2018) or other bias (Fehrenbacher et al., 2016; Herbst et al., 2016; Fehrenbacher et al., 2018; Barlesi et al., 2019).

### Efficacy

#### Overall Survival

**FIGURE 3:**
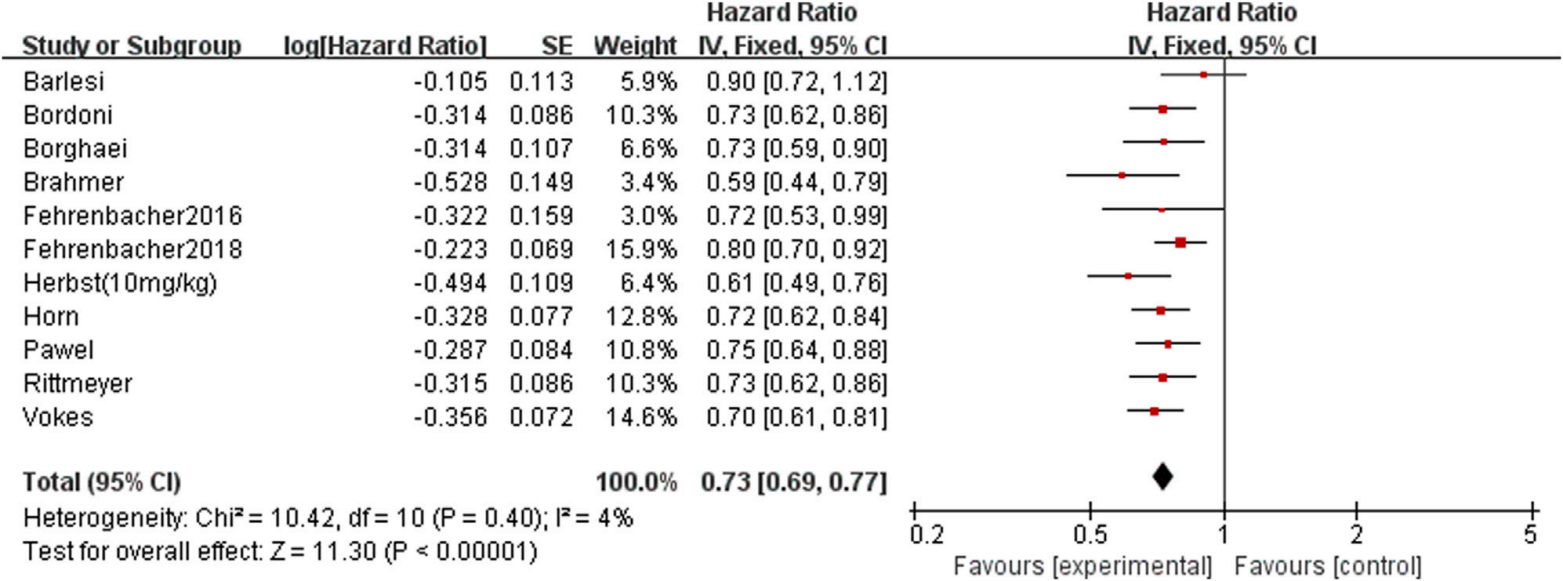
Comparison of overall survival between groups.

**FIGURE 4:**
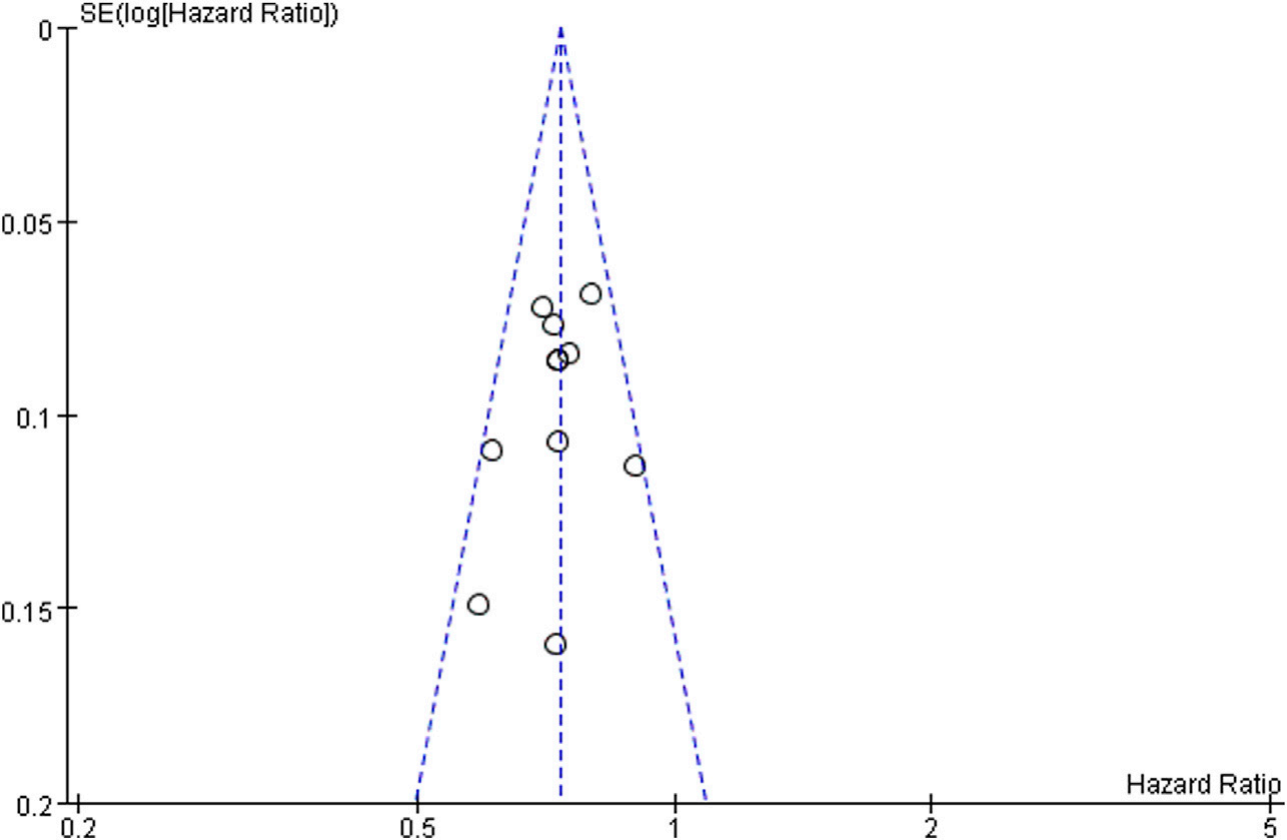
Funnel chart of comparison of overall survival.

There have 11 RCTs reported overall survival, and there was no statistical heterogeneity among the studies. The meta-analysis results suggest that the overall survival of the PD-1/PD-L1 group is higher than docetaxel group. The difference between groups is statistically significant (HR = 0.73, 95%CI:0.69∼0.77, *p* < 0.00001), it suggests that the efficacy of PD-1/PD-L1 in the treatment of NSCLC is better than docetaxel chemotherapy (Figure 3 Comparison of overall survival between groups). Funnel chart is shown in Figure 4 (Funnel chart of comparison of overall survival), no obvious publication bias.

#### Progression-free Survival

**FIGURE 5:**
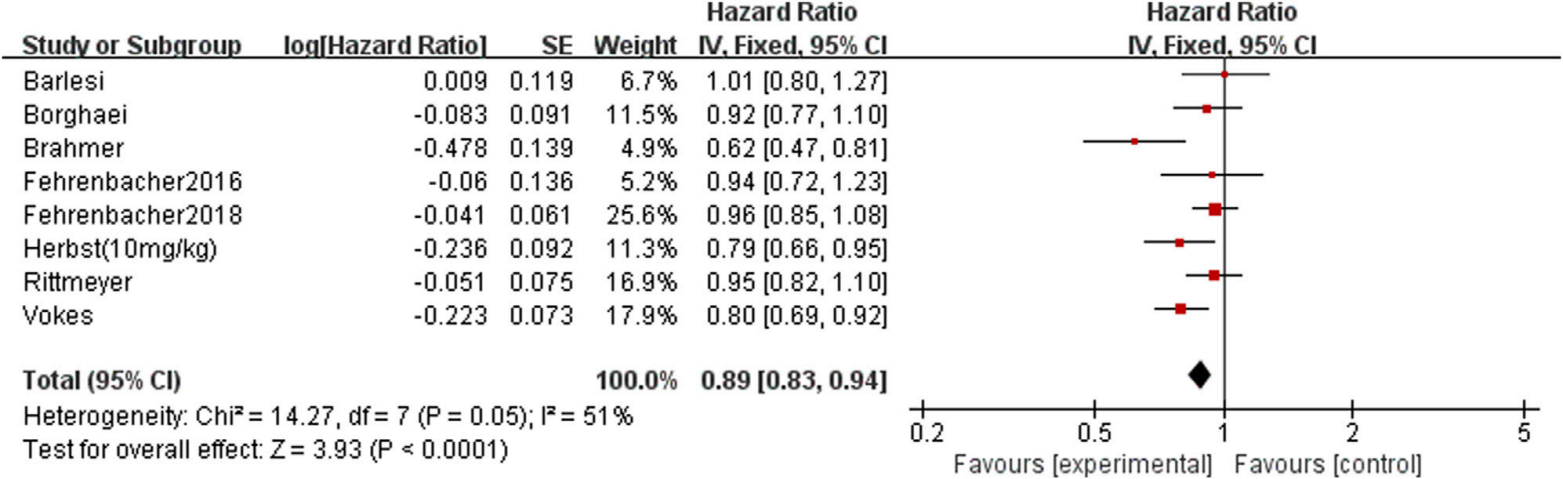
Comparison of progression-free survival between groups.

There have eight RCTs reported progression-free survival, and there was no significant statistical heterogeneity among the studies. The meta-analysis results suggest that the progression-free survival of the PD-1/PD-L1 group is higher than docetaxel group. The difference between groups is statistically significant (HR = 0.89, 95%CI: 0.83∼0.94, *p* < 0.00001), it suggests that the efficacy of PD-1/PD-L1 in the treatment of NSCLC is better than docetaxel chemotherapy (Figure 5 Comparison of progression-free survival between groups).

#### Safety

**FIGURE 6:**
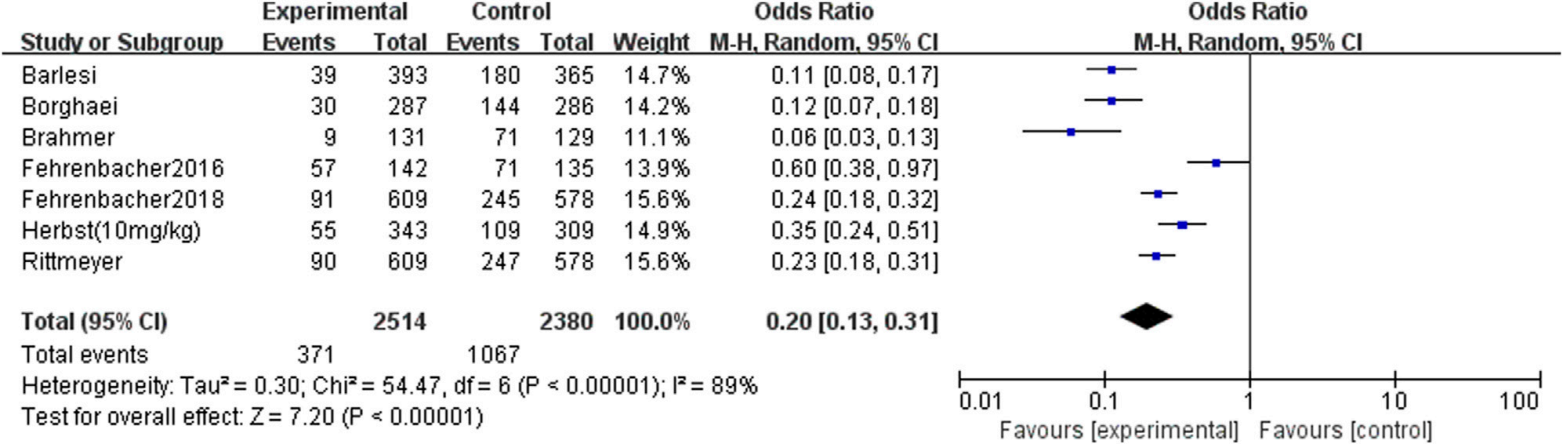
Comparison of treatment-related grade 3 or higher adverse events between groups.

There have only seven RCTs reported the incidence of treatment-related grade 3 or higher adverse events, and the difference was statistically significant (OR = 0.20, 95% CI: 0.13∼0.31, *p* < 0.00001). The risk of the serious adverse reactions in the PD-1/PD-L1 treatment group was lower than the docetaxel group, suggesting that the use of docetaxel may increase the additional treatment burden of patients (Figure 6 Comparison of treatment-related grade 3 or higher adverse events between groups).

#### Publication Bias Test and Sensitivity Analysis

Publication bias analysis was performed only in overall survival because other analysis included fewer than 10 studies, and the funnel graph is symmetrical, showed no obvious publication bias. Sensitivity analysis was performed on the results, the influence of each study was examined by repeating meta-analyses when each study was omitted, but no significant change was observed, indicating that the results of this study were stable.

## Discussion

Lung cancer is the most common malignant tumor in clinic, which seriously threatens people’s health. Immunotherapy with immune microenvironment intervention as the core strategy has developed rapidly and has become a hot spot in the treatment of lung cancer. Immunotherapy drugs used for lung cancer treatment are mainly immune checkpoint inhibitors, which can restore the body’s anti-tumor immune response, thereby killing tumors. Based on the molecular pathology detection of NSCLC, the corresponding drugs are selected for precise treatment according to the expression levels of different molecules. Immunotherapy has obvious advantages in prolonging the survival period of NSCLC. The effective rate of chemotherapy for patients with NSCLC is only 8–9%. With the development of immunotherapy, there have been many clinical trials, such as Brahmer et al. 2015, Borghaei et al. 2015, Herbst et al. 2016 and Gadgeel et al. 2019 have confirmed that Nivolumab, Pembrolizumab, and Atezolizumab significantly prolong overall survival (OS) compared with chemotherapy.

This article reviews the overall survival and progression-free survival of all currently published randomized controlled trials in patients with NSCLC treated with PD-1/PD-L1 drugs and docetaxel-containing chemotherapy regimens. The results of the analysis showed that patients who received PD-1/PD-L1 had better results than those who received docetaxel chemotherapy [overall survival: (HR = 0.73, 95%CI: 0.69∼0.77, *p* < 0.00001), progression-free survival (HR = 0.89, 95%CI: 0.83∼0.94, *p* < 0.00001)]; PD-1/PD-L1 significantly prolonged overall survival and progression-free survival than docetaxel. Although this meta-analysis included three drugs with similar mechanisms of action (Atezolizumab, Nivolumab and Pembrolizumab), but as far as the overall results were concerned, they were not found to be different. Atezolizumab is an engineering humanized monoclonal antibody targeting PD-L1 (Gutzmer et al., 2020). It could eliminate the antibody dependent cytotoxicity and inhibit the consumption of activated T cells by modifying the crystal fragment domain (Lee et al., 2020). Nivolumab is a humanized IgG4 monoclonal antibody that inhibits PD-1 receptor (Koppolu and Rekha Vasigala, 2018). It is expressed in activated CD4 positive and CD8 positive T cells, NK cells, B cells and monocytes, as well as in some tumor cells and tumor infiltrating lymphocytes. It binds to PD-1 receptor and releases the inhibition of immune response mediated by PD-1 pathway, including anti-tumor immune response (Gao and McDermott, 2018). Pembrolizumab is a humanized anti-PD-1 monoclonal antibody, which could enhance the anti-tumor immune activity. It was approved by the US Food and drug administration to be listed in the United States since 2015 (Kwok et al., 2016). In NCCN guidelines, Pembrolizumab has been recommended as the first-line treatment for non-small cell lung cancer (McDermott and Jimeno, 2015). At the same time, seven randomized controlled trials reported that docetaxel was more toxic than PD-1/PD-L1 [treatment-related grade 3 or higher adverse events: (OR = 0.20, 95% CI: 0.13∼0.31, *p* < 0.00001)]; compared with docetaxel, PD-1/PD-L1 with lower treatment-related grade 3 or higher adverse events, especially with lower risk of myelosuppression, gastrointestinal reactions and other adverse reactions. PD-1 and its ligand PD-L1 were negative costimulatory factors, belonging to B7 family immunoglobulins, which played an important role in the activation of T cells (Sunshine and Taube, 2015). The highly expressed PD-1 specifically combined with PD-L1 to provide inhibitory signals, inhibited the activation and proliferation of T cells, and then induced T cell apoptosis to form an immunosuppressive microenvironment (Xia et al., 2019).

In recent years, immune checkpoint inhibitors, especially PD-1 and its ligand PD-L1 inhibitors, have been widely concerned because of their universality, significant anti-tumor activity and good safety, which improve the prognosis of patients with advanced non-small cell lung cancer. PD-1 is expressed in various immune cells, including activated T lymphocytes, B lymphocytes, macrophages and dendritic cells, as well as non lymphocytes or tissues. The interaction between PD-1 and PD-L1 is common in NSCLC, which can down regulate T cell activation and promote tumor immune escape. Anti PD-1/PD-L1 therapy uses PD-1/PD-L1 immune checkpoint inhibitor antibodies to interfere with PD-1/PD-L1 mediated signal transduction to restore anti-tumor immunity. Immunotherapy showed a long-term clinical response, which significantly improved the progression-free survival and overall survival of local and metastatic NSCLC. The advantage of immunotherapy alone is that once patients respond, the response effect is often lasting, even after a few years, and the incidence of acquired drug resistance is low.

Immunotherapy and targeted therapy are the models of clinical implementation of precision medicine, which embody the concept of transforming from tumor cell centered to tumor growth microenvironment, and achieve a breakthrough in the efficacy of solid tumors to a certain extent. With the development of more and more clinical trials of immunotherapy, researchers pay more attention to its adverse reactions. The common adverse reactions of immunotherapy include various immune inflammation, fever, fatigue, skin itching, rash, thrombocytopenia, liver and kidney dysfunction, especially fatal myocarditis and pulmonary toxicity. Considering the possible adverse reactions and economic burden in immunotherapy, it is of great clinical significance to find biomarkers that can accurately predict the response to immunotherapy, which is also the biggest problem in immunotherapy of NSCLC at this stage. At present, the existing biomarkers have certain value in predicting the prognosis and curative effect of lung cancer patients, but they all have limitations and shortcomings. It is necessary to develop more effective biomarkers to optimize the interests of patients and guide treatment. Therefore, this field is worthy of further research and exploration.

This study had some limitations. First, we conducted a comprehensive search on the corresponding database, and only included 12 studies for systematic reviews and meta-analysis. The amount of literature is too small, which may bias the interpretation of the results. Second, there is obvious heterogeneity in some comparisons, which may affect the reliability of the results. Third, some of the included studies did not report data such as overall survival, progression-free survival and treatment-related grade 3 or higher adverse events, so statistical analysis could not be performed. Although there are some limitations in the included studies, this study was reported strictly in accordance with the PRISMA items in order to reduce the bias as much as possible.

In conclusion, the anti-PD-1/PD-L1 Inhibitors has certain advantages in effectiveness compared to docetaxel, lower incidence of treatment-related grade 3 or higher adverse events. The results still need to be confirmed by a multi-center, large sample, and high-quality research.

## Data Availability

The original contributions presented in the study are included in the article/Supplementary Material, further inquiries can be directed to the corresponding author.
